# How authority affects social evaluations of negotiation words

**DOI:** 10.1186/s41235-025-00669-8

**Published:** 2025-09-01

**Authors:** Allison Nguyen, Jean E. Fox Tree

**Affiliations:** 1 Department of Psychology, University of California, 1156 High St, Santa Cruz, CA 95064 USA; 2 Department of Psychology, DeGarmo Hall, Illinois State University, 100 N University St, Normal, IL 61761 USA

**Keywords:** Negotiation words, Hedges and boosters, Speaker authority, *Totally*, Discourse processes, Online communication

## Abstract

Understanding how authority affects social evaluation of written communication is crucial for understanding how online communication technologies can be effectively deployed. We examined how negotiation words affected perceived ratings of knowledgeableness, professionalism, politeness, and friendliness across three levels of authority (professor, teaching assistant, student) while asking and answering questions in a mock online forum. The greatest distinction across groups was in professionalism. For professors and TAs, most negotiation words lowered professionalism, but this was not the case for students. The greatest similarity across groups was for the words *clearly* and *obviously*. Both made people appear less friendly and less polite. Compared to the unmodified condition, hedges (e.g., kinda) decreased knowledgeableness but boosters (e.g., absolutely) did not increase knowledgeableness. One negotiation word, *totally*, had a surprising pattern—it helped higher authority speakers appear more friendly.

## Introduction

Modern higher education settings involve many communicative media that differ from those professors grew up with, such as chat groups linked to courses. These groups can be officially sanctioned, with instructors present on the site to guide discussion or provide answers to questions, or they can be unofficial, with students supporting each other away from the eyes of their instructors. A critical factor to explore in officially supported chats is how the presentation of authority affects social evaluations of *negotiation words*.

Negotiation words are words like *kinda, sorta, basically,* and *absolutely* that indicate the level of looseness or certainty of the ideas expressed. The level of commitment provides important information to conversational participants not only about the topic being discussed (a *kind of indie pop concert* is different from a *totally indie pop concert*; Nguyen & Fox Tree, under review, p. 25) but also about the way the speaker or writer feels about the topic being discussed (a person who uses *kind of* might be claiming lack of knowledge or distancing from a commitment to their words). These *negotiation words* fall on a continuum from lack of sureness and certainty (*sorta*, *sort of*, *kinda*, *kind of*) through mid-way words (*basically*) to words expressing sureness and certainty (*absolutely*, *certainly*, *totally*, *clearly*, *obviously*; Nguyen & Fox Tree, under review). This continuum was determined by having participants rate these words in and out of context on sureness and certainty (please see Nguyen & Fox Tree, under review, for details). The less sure words cluster together and form what are considered *hedges* in communication and the more sure words cluster together and form what are considered *boosters* in communication (Hyland, 2000; Hyland, 1996, 1998, 2000, 2005; Jalilifar & Alavi-Nia, 2012; Zou & Hyland, 2023; Vázquez Orta & Giner, 2008; 2009; Holmes, 1990; Kashiha, 2022). We selected hedges and boosters based on prior literature (see Nguyen & Fox Tree, under review, for a detailed discussion of these selections).

Hedges are short phrases or words that speakers can use to pragmatically mark information. The marking may indicate stance (e.g., Hu & Cao, 2011) or uncertainty (e.g., Kashiha, 2022), or may pragmatically highlight information (e.g., Hyland, 1996). Hedges can also indicate an estimation, such as in expressing quantities (Liu & Fox Tree, 2012). In a study of how much negotiation versus telling boosters conveyed, hedges clustered together as words that were high in negotiation but low in telling (Nguyen & Fox Tree, under review). However, not all participants rated them as maximally low on telling, and there was a range across hedges.

Boosters, like hedges, can be used to indicate stance (e.g., Hu & Cao, 2011), or pragmatically highlight information (e.g., Hyland, 1996). However, instead of marking uncertainty, boosters have been largely assumed to indicate certainty, or as words that intensify or maximize meaning (Beltrama & Staum Casasanto, 2017; Ito & Tagliamonte, 2003). While these words may be low in negotiation, it is likely that they are not free from negotiation. Speakers may deliberately choose these words to indicate that they are willing to accommodate a small amount of negotiation. In a study of how much negotiation versus telling boosters conveyed, boosters clustered together as words that were low in negotiation and high in telling, but they were not maximally telling—not all participants rated any one booster as maximally high on telling, and there was a range across boosters (Nguyen & Fox Tree, under review).

While the categories of hedge and booster are not the only ways to divide these words (Holmes, 1984; Ito & Tagliamonte, 2003; Lakoff, 1973), we believe the categories describe endpoints of a continuum that signal to addressees what amount of negotiation the speaker is willing to entertain. This means that both boosters and hedges not only represent information about the world itself, but also the speaker’s knowledge states. The label negotiation words captures this. Other phenomena in spoken speech can also be categorized in multiple ways. For example, the label backchannels is a broad category that can be divided into many different subcategories, such as vertical or horizontal, or generic and specific, where each subcategory serves a different function and may or may not overlap with other categories (Bangerter & Clark, 2003; Fox Tree et al., 2021; Nguyen et al., 2024).

Because speakers deliberately choose to signal negotiation to their addresses in order to ground, it is likely that there are words in both the hedge and the booster groupings that otherwise would not form a natural class. For example, in the study of negotiation versus telling, there were in-between words like *pretty* and *basically* that were somewhat negotiating, somewhat telling (Nguyen & Fox Tree, under review). This approach differs from what we could call an intensifier approach where some of these words (boosters) are conceptualized as pushing the literal meaning to the highest extreme (Beltrama, 2018; Ito & Tagliamonte, 2003). In an intensifier approach, *very tall* and *absolutely tall* meaning “as tall as one could possibly be.” This approach is concerned with the scale these words sit on. In contrast, we focus on the negotiation between individuals. Our approach acknowledges that these are intensifiers, but suggests that they might function metalinguistically in different ways—deliberately choosing *very* means something different from choosing *absolutely*.

Another factor to consider is historical changes in the uses of the words. Negotiation words vary in popularity over time and by subcultures (Ito & Tagliamonte, 2003). For example, in a study of language used in York, England, in around 1998, *really* was more common with young people than *very* (Ito & Tagliamonte, 2003), and in general, younger speakers used boosters more frequently than older speakers (Tagliamonte, 2008). The amount of negotiation conveyed by different words may similarly change over time or by speaker groups. As an analogic example, the word *literally* has been changing to mean *metaphorically* (as in “This book literally blew my mind,” Kostadinova, 2018, p. 29). Were a set of words identified to express literalness, *literally* may be less literal than *precisely* or *indisputably*.

In this report, we examined how negotiation words affected perceived ratings of knowledgeableness, professionalism, politeness, and friendliness across three levels of authority (professor, teaching assistant, student) while asking and answering questions in a mock online forum. These features were assessed because they are relevant to our setting of instructors in an educational mock Discord setting. Knowledgeable, professional, polite, and friendly instructors can be understood to be better instructors. Each of these characteristics may vary by status. For example, professors may be more knowledgeable and professional than TAs and peers, TAs may be more polite than professors and peers, and peers may be more friendly than professors or TAs.

### Authority in text

Language use occurs in contexts, and this includes not only the settings in which the communication occurs (such as at a workplace or at home, or via different modalities of communication, such as spoken or written) but the speakers themselves. Speakers use information readily available to them through observing their partners to make judgements, including how attractive they find their conversational partner (Michalsky & Schoormann, 2021; Störmer & Alvarez, 2016), whether their partner is dressed appropriately (Brem & Niebuhr, 2021; Feinberg et al., 1992; Küster et al., 2019), and how much they like their partner—or their partner likes them (Boothby et al., 2018). Other judgements speakers might make include estimations of socioeconomic status, gender, age, and authority or power (e.g., Hester & Hehman, 2023; Livingston & Pearce, 2009; Rhodes, 2009).

Speakers in conversations also have access to *sociolinguistic cues* (gendered language assumptions, speech patterns, phonetic information, and words unique to specific dialects) that provide information about the speaker beyond the words they are using (Cocchiara, et. al., 2016; Rubin & Greene, 1992; Holmes, 1990; Lakoff, 1973; Schötz, 2007). Like visual cues, language use can provide cues about where we grew up, who we are, and what groups we identify with. These cues are also used when we evaluate speakers, and by extension, the information they provide.

Sociolinguistic cues can affect social evaluations of authority (Alavi-Nia & Jalilifar, 2013; Bucholtz, 2001; Campbell-Kibler, 2009; Hilton & Jeong, 2019; Vornik et al., 2003). For example, speakers who produce the full ending *ing* in words such as *walking* are perceived as more educated, and less likely to be working-class, compared to speakers who produce *in’*, as in *walkin’* (Campbell-Kibler, 2009). Other research in socio-phonetics has shown similar findings, with /t/ released as a stop rather than as a tap indicative of nerd culture (Bucholtz, 2001). When used in political speech, the stop can be interpreted as more authoritative (Podesva et al., 2015). At a broader level, accents in general are tied to levels of social power, with more highly ranked accents suggesting higher power (Vornik et al., 2003). Even hand gestures used by speakers can influence what viewers think of the speaker, including their persuasiveness (Maricchiolo et al., 2020). Sociolinguistic cues that correlate to power and social attractiveness can lead people to believe information, even if that information is false (Vornik et al., 2003), and in the case of political speech, can lead to less critical evaluations of policy (Bi et al., 2021).

While these studies were conducted on spoken discourse, it is likely that the same effects happen in written discourse. For example, we know that using more formal writing, such as periods, makes text feel less hyperpartisan (Nguyen et al., 2022), which can be related to feeling more authoritative. We also know that feeling more authoritative influences how people text; people who felt more power were less likely to respond to non-standard writing (such as extra vowels or punctuation marks) with non-standard writing (Adams et al., 2018). Sociolinguistic cues are readily available not only in face-to-face speech but also in writing (see Farrokhi & Emami, 2008; Ho, 2018; Nguyen & Fox Tree, under review; Vázquez Orta & Giner, 2008; 2009).

One way to express authority in writing is with boosters and hedges. Boosters have been found in .4% to 1.1% of academic papers (Farrokhi & Emami, 2008; Ho, 2018; Vázquez Orta & Giner, 2009). Boosters have also been found to occur at the rate of .8% in emails (Ho, 2018) and from less than .001% to .02% in instant messages (Nguyen & Fox Tree, under review). Hedges occur in .07% to 2.8% of academic papers (Farrokhi & Emami, 2008; Vazquez & Giner, 2008). Hedges have been also found to occur at the rate of 1.1% in emails (Ho, 2018) and from less than .001% to .2% in instant messages (Nguyen & Fox Tree, under review). These rates may seem low. However, low rates of phenomena have been shown to systematically vary across different types of communication (such as hyperpartisan and nonhyperpartisan communication) and have been argued to make a difference in how people feel about the communication, such as creating feelings of closeness between the writer and reader (Nguyen et al., 2022).

While expressions of authority using morphological structures (adding *ing*) and words (negotiation words) are common in writing, in online spaces, sociolinguistic cues can also take on visual forms, to emulate the visual cues available to us in face-to-face communication. There are many ways an individual can choose to represent themselves (or not) in these online spaces, from emoji skin tone (Robertson et al., 2018, 2021), where people can choose a skin tone close to their own, or keep the default yellow, to profile photographs (Herring & Kapidzic, 2015; Manago, et al., 2008), where people can carefully curate the image of themselves they want to present to the world. Profile pictures, usernames, gifs, and emoji skin tones can make up for limited information communicators may have about the appearance of their interlocutors. Importantly, these sociolinguistic cues are assumed to be based on the actual communicator (Robertson, 2018; Robertson, 2021; Herring & Kapidzic, 2015; Manago et al., 2008; Tolins & Samermit, 2016). This means that speakers adapt to what they have and use emoji skin tone, profile photographs, usernames, biography descriptions, and word choices as cues to make inferences about their interlocutors.

Formality in speech often varies with social distance. For example, speakers with low social distance (classmates) are likely to speak informally and speakers with high social distance (professors and students) are likely to speak more formally with each other (Koppen et al., 2019). However, the speech conventions on Discord are informal. Thus, even though the speakers likely carry different levels of authority, the informalness of the platform and the lack of information about the speakers beyond their title might act as an equalizer, lowering all the standards of speech to what is appropriate for the platform.

In the current study, we used a mock online group discussion platform (Discord) as the framing device. We focused on verbal negotiation words and did not include visual information (such as skin tones or emojis). Nonetheless, we believe that the frequent co-occurrence of multiple sociolinguistic features on Discord in non-laboratory, real-life settings would help us highlight the sociolinguistic aspects of negotiation words in online communication. Said another way, because Discord is often imbued with sociolinguistic information when it’s used in daily life, it was a good vehicle for our assessment of the sociolinguistic information conveyed by negotiation words.

### Online question asking and answering forums

The norms of written spontaneous communication are evolving rapidly. Professors, TAs, and classmates all interacting together in a fairly anonymous online space took off in earnest as a response to the challenges of educating during a pandemic. The creation of online spaces has continued post-pandemic, with some spaces existing as sanctioned classroom extensions and some arising under the leadership of enterprising students. This results in a wide variety of spaces, some with professors and teaching assistants active on them, some with professors and teaching assistants on them but not active, and some without professors and teaching assistants. Discord, which started as a platform for gamers, was co-opted to extend the classroom, and it has been shown that it creates a closer, more interactive environment than either entirely in-person or entirely online classes (Wiles & Simmons, 2022). Environments where people feel closer and casual might lead to more spontaneous speech features like hedges (similar to how hyperpartisan spaces also have more spontaneous speech features—see Nguyen, et al., 2022, for more).

Written communication norms have long been shifting to favor spoken norms. This shift was noticed even before texting was ubiquitous (Baron, 1984). Predictably, greater experience with texting was related to the use of more spoken-like phenomena (Fox Tree et al., 2011). While some written communication platforms can have formal expectations, such as email communications to a professor (Marjanović, 2017), some facilitate informal communication over formal communication. So it would not be a surprise if even authority figures produced writing that felt casual and spontaneous on platforms that are informal. A key feature of informal communication is the use of words that in former times were primarily spoken, not written, including hedges (Vázquez Orta & Giner, 2008; 2009), discourse markers such as *oh*, *so*, and *like* (Fox Tree et al., 2011; Guydish et al., 2024), *um*s and *uh*s (D’Arcey et al., 2019), and *okay* and *right* (Nguyen et al., 2024). This transformation likely results from our moving into an era where much of people’s daily communication is mediated through online platforms.

### Current experiment

To test whether speaker factors change how negotiation words are interpreted, we manipulated levels of authority (low, medium, high) in a mock online forum for asking and answering questions modeled after Discord. We asked whether a professor’s use of negotiation words implied something different from a teaching assistant’s (TA’s), or from an undergraduate student’s. By setting the experiment in an online chat platform where real names (and other information like gender and race) are often not accessible, we are able to look at how speaker information affects social evaluation with a high amount of control over what readers know about the speaker. We note here that Discord became commonly used during the pandemic as a platform for extending classrooms (Wiles & Simmons, 2022), and participants were familiar with the idea that professors, TAs, and students would have interactions like the ones we presented.

The usual understanding of boosters and hedges reflects the knowledge state of the speaker (Hyland, 1998), and accordingly, we predicted that boosters will increase perceived knowledgeableness of the speaker and hedges will decrease knowledgeableness, regardless of how much authority the producer of the negotiation word has. For the other three social dimensions (politeness, friendliness, and professionalism), we hypothesized that boosters would increase distance between interlocutors because they imply less negotiation (Nguyen & Fox Tree, under review). Hedges, on the other hand, would decrease distance because they imply more negotiation (Nguyen & Fox Tree, under review). Consequently, we hypothesized that more distance would be less friendly and more professional and that less distance would be more friendly and less professional. Distance alone is not differentially predictive for politeness because increasing distance can indicate deference (negative politeness) and decreasing distance can indicate collegiality (positive politeness; Brown & Levinson, 2011). However, because they imply telling rather than negotiating (Nguyen & Fox Tree, under review), we predicted that boosters will indicate less politeness than hedges.

## Method

University students read a texted exchange about a cognitive psychology topic and provided open-ended responses as well as ratings of knowledgeableness, friendliness, politeness, and professionalism. The texted exchange only varied by the negotiation words used in the exchange.

### Participants

There were 245 participants from a West Coast research university subject pool. Participants ranged in age from 18 to 47 (mean age = 19.8) and received course credit for participating. 174 self-identified as women, 52 as men, and 14 as genderqueer or nonbinary. Participants were primarily from the state of California and were overwhelmingly from a suburban area (suburban = 137, urban = 91, rural = 17).

### Materials

Participants saw a factual passage about fMRIs (adapted from *How FMRI works*, The Open University 2024) as a response to a question posed by a student. This passage was selected due to topic—it was expected that participants would be familiar with the term fMRI but have little to no experience with how the technology works.

Participants saw a framing sentence before each constructed stimulus. The sentence was always “A student is messaging their professor on Discord” for the professor condition, “A student is messaging their TA on Discord.” for the TA condition, and “A student is messaging their classmate on Discord.” for the student condition. Following the framing sentence, participants saw the constructed dialogue, which was always formatted in the following manner:

1. Student: I have often wondered about fMRI technology and other kinds of black box tech, like airport scanners or x ray machines. What is fMRI technology and how does it work?
2. Professor: Functional magnetic resonance imaging, or FMRI, [inserted word location] works by detecting the changes in blood oxygenation and flow that occur in response to neural activity—when a brain area is more active it consumes more oxygen and to meet this increased demand blood flow increases to the active area.

The label “Professor” was switched for “TA” or “Classmate” depending on condition. Where *inserted word location* is noted in (2), either nothing was inserted (the unmodified conditions) or a word from the following 11 words was inserted: *absolutely*, *basically*, *certainly*, *clearly*, *kinda*, *kind of*, *obviously*, *partially*, *sorta*, *sort of*, *totally*. These constructed paragraphs were normed and rated acceptable.

In open response questions, participants referred to Discord, suggesting they recognized the dialogues as in Discord chat format.

### Design

The experiment used a 3-level (high, medium, low authority) between-participants design. The *high-authority* condition consisted of the dialogue occurring between a professor and student. The *medium-authority* condition had the dialogue occurring between a teaching assistant and student. TAs were chosen because the participants would understand TAs to have less authority than professors in the classroom but more authority than classmates. The *same-authority* condition was two students participating in the dialogue. Participants were randomly sorted into conditions.

### Procedure

Participants saw a passage about fMRIs and completed 12 trials (one for each word of negotiation, and one unmodified version). The passage was held constant throughout each trial with only the target word varying. However, participants were told to imagine a new set of speakers each time, and all 12 trials were randomized across participants. Participants were allowed to spend as much time as they wanted reading the passage. Keeping the passage consistent between trials focused judgements on the items of interest, the negotiation words. All other elements of the passage—the topic, the word choices, the length—remained the same.

After reading each passage, participants answered a series of questions, with the passage still in view. Three were free-response, asking for opinions of the question answerer (professor, TA, or classmate) and the question asker (student), as well as why the participants felt the way they did. Answers to these questions will not be discussed in this report. Four questions were closed questions presented on 5-point scales: (1) *How knowledgeable do you think the professor is?* (on a scale from 1: Not knowledgeable at all to 5: Extremely knowledgeable), (2) *How friendly do you think the professor is?* (on a scale from 1: Not friendly to 5: Extremely friendly), (3) *How polite do you think the professor is?* (on a scale from 1: Not polite to 5: Extremely polite), and (4) *How professional do you think the professor is?* (on a scale from 1: Extremely unprofessional to 5: Extremely professional). The same measures were also collected for the student, though these were not examined (as the student’s question in the prompt never varied).

## Results

We discuss the closed question responses below. Results are discussed by knowledgeableness, friendliness, politeness, and professionalism.

### Knowledgeableness

Looking at the unmodified passage across the three conditions (professor, TA, classmate), there was no difference between perceptions of knowledgeableness, *χ*^*2*^(2, N = 245) = 5.717, *p* = .05. Professors scored an average of 3.97 (SD = 1.10), TAs an average of 3.93 (SD = 1.02), and classmates an average of 3.47 (SD = 1.5).

The rating given to each speaker when the answer was modified by a word was examined using a nonparametric Kruskal–Wallis test. No significant differences were found between authority conditions for any of the words tested. While authority did not seem to affect how speakers were﻿ perceived, it is possible that within each condition, words might pattern differently in perceived knowledge. To examine within-condition results, nonparametric Friedman’s tests were used.

#### Professor knowledgeableness

Within the professor condition (high authority), words differed from each other (*χ*^*2*^(11) = 190.62, *p* < .001). Pairwise comparisons with Bonferroni corrections are presented in Table 1.

**Table 1:** Pairwise comparisons for professor knowledgeableness ratings from 1 (Not knowledgeable at all) to 5 (Extremely knowledgeable)

Word	Mean (SD)	Differs from these Words	Does not Differ from these Words
unmodified	3.93 (1.10)	kinda****, kind of ****, sorta****, sort of****, totally**	absolutely, basically, certainly, clearly, obviously, partially
absolutely	3.57 (1.49)	kinda****, kind of****, sorta****, sort of****	unmodified, basically, certainly, clearly, obviously, partially, totally
basically	3.69 (1.24)	kinda****, kind of****, sorta****, sort of****	unmodified, absolutely, certainly, clearly, obviously, partially, totally
certainly	3.53 (1.48)	kinda****, kind of***, sorta****, sort of**	unmodified, absolutely, basically, clearly, obviously, partially, totally
clearly	3.69 (1.36)	kinda****, kind of****, sorta****, sort of****	unmodified, absolutely, basically, certainly, obviously, partially, totally
kinda	2.83 (1.06)	unmodified****, absolutely****, basically****, certainly****, clearly****, obviously****, partially**, totally*	kind of, sorta, sort of
kind of	2.77 (1.33)	unmodified****, absolutely****, basically****, certainly***, clearly****, obviously**	kinda, partially, sort of, sorta, totally
obviously	3.53 (1.40)	kinda****, kind of**, sorta****, sort of**	unmodified, absolutely, basically, certainly, clearly, partially, totally
partially	3.47 (1.07)	kinda**, sorta**	unmodified, absolutely, basically, certainly, clearly, kind of, obviously, sort of, totally
sorta	2.75 (1.15)	unmodified****, absolutely****, basically****, certainly****, clearly****, obviously****, partially**, totally*	kinda, kind of, sort of
sort of	2.97 (1.17)	unmodified****, absolutely****, basically****, certainly** clearly****, obviously**	kinda, kind of, partially, totally, sorta,
totally	3.36 (1.18)	unmodified**, kinda*, sorta*	absolutely, basically, certainly, clearly, kind of, obviously, partially, sort of

Asterisks indicate significance level (* = .05; ** = .01; *** = .001, **** = .0001)

Hedges (*kinda, kind of, sorta,* and *sort of*) had lower ratings of perceived knowledge compared to the *unmodified* condition, showing that they do act as markers of uncertainty that observers can pick up on. As seen previously (Nguyen & Fox Tree, under review), these four hedges clustered together, with no differences between the four words in affecting perceived knowledgeableness. Boosters (*absolutely, obviously, clearly, certainly* and *basically*) also clustered together (we note that *basically* clustered with boosters for professor knowledgeableness, which differs from what was observed in prior studies where *basically* differed from boosters in ratings of negotiation versus telling; see Nguyen & Fox Tree, under review). Boosters did not differ from the unmodified condition; that is, they did not boost perceived knowledge compared to saying nothing. But they did increase perceptions of knowledge compared to hedges.

An unusual finding was that *totally* behaved like a booster and like a hedge. It did not differ from the boosters *absolutely, obviously, clearly, certainly* and it did not differ from the hedges *kind of* and *sort of*. *Totally* was perceived as less knowledgeable than the unmodified condition, further supporting its potential use as a hedge in circumstances where interlocutors are trying to judge speaker knowledge. This is likely tied to other social cues associated with *totally*—in some cases, *totally* can indicate a speaker who is female-coded, younger, and lower in authority (Beltrama & Staum Casasanto, 2017). Gender, age, and authority can all affect social perceptions of knowledgeableness. So, one possibility is that *totally* made addressees think of these factors and that these factors in turn affected judgements of knowledgeableness. Totally was perceived as more knowledgeable than *kinda* and *sorta*, however. *Partially* behaved like *totally* except that it did not affect ratings compared to the unmodified condition.

#### TA knowledgeableness

Within the student-TA condition (medium authority), several words differed from each other, *χ*^*2*^(11) = 211.88, *p* < .001. Pairwise comparisons with Bonferroni corrections are presented in Table 2.

**Table 2:** Pairwise comparisons for TA knowledgeableness ratings from 1 (Not knowledgeable at all) to 5 (Extremely knowledgeable)

Word	Mean (SD)	Differs from these Words	Does not Differ from these Words
unmodified	3.93 (1.02)	kinda****, kind of****, partially**, sorta****, sort of****, totally**	absolutely, basically, certainly, clearly, obviously
absolutely	3.87 (.80)	kinda****, kind of****, sorta****, sort of****	unmodified, basically, certainly, clearly, obviously, partially, totally
basically	3.63 (.99)	kinda****, kind of****, sorta****, sort of****	unmodified, absolutely, certainly, clearly, obviously, partially, totally
certainly	3.82 (1.04)	kinda****, kind of****, sorta****, sort of****	unmodified, absolutely, basically, clearly, obviously, partially, totally
clearly	3.54 (1.08)	kinda***, kind of***, sorta****, sort of****	unmodified, basically, certainly, absolutely, partially, totally, obviously
kinda	2.92 (1.08)	unmodified****, absolutely****, basically****, certainly****, clearly***, obviously**	kind of, partially, sorta, sort of, totally
kind of	2.82 (1.27)	unmodified****, absolutely****, basically****, certainly****, clearly***, obviously**	kinda, partially, sorta, sort of, totally
obviously	3.49 (1.18)	kinda**, kind of**, sorta***, sort of***	unmodified, absolutely, basically, certainly, clearly, partially, totally
partially	3.38 (1.12)	unmodified**	absolutely, basically, certainly, clearly, kinda, kind of, obviously, sorta, sort of, totally
sorta	2.89 (1.03)	unmodified****, absolutely****, basically****, clearly****, certainly****, obviously***, totally*	kinda, kind of, partially, sort of
sort of	2.85 (1.14)	unmodified****, absolutely****, basically****, certainly****, clearly****, obviously***, totally*	kinda, kind of, partially, sorta
totally	3.36 (1.15)	unmodified**, sorta*, sort of*	absolutely, basically, certainly, clearly, kinda, kind of, obviously, partially

Asterisks indicate significance level (* = .05; ** = .01; *** = .001, **** = .0001)

Like the high-authority professor condition, the same groups emerge. Hedges (*kinda*, *kind of, sorta*, and *sort of*) had lower ratings of perceived knowledge compared to boosters (*absolutely, obviously, clearly, certainly* and *basically*; we note that as with professor knowledgeableness, *basically* clustered with boosters for TA knowledgeableness, which differs from what was observed in prior studies where *basically* differed from boosters in ratings of negotiation versus telling; see Nguyen & Fox Tree, under review). Hedges also lowered perceptions of perceived knowledge compared to the *unmodified* condition. So, boosters boost in comparison to hedges. However, they do *not* boost perceptions compared to the *unmodified* condition, again. Finally, *totally* here acts as a booster but only in comparison with *sorta* and *sort of*, and *partially* only lowers perceptions compared to the *unmodified* condition.

Once again, *totally* behaved like a booster and like a hedge. It did not differ from the boosters *absolutely, obviously, clearly,* and *certainly*, and it did not differ from the hedges *kinda* and *kind of*. *Totally* was perceived as less knowledgeable than the *unmodified* condition, further supporting its potential use as a hedge. It was more knowledgeable than *sorta* and *sort of*, however. *Partially* behaved like *totally* except that it did not differ from *sorta* and *sort of*.

#### Classmate knowledgeableness

Within the student-classmate condition (same-level authority), several words differed from each other, *χ*^*2*^(11) = 144.58, *p* < .001. Pairwise comparisons between groups were carried out. Table 3 breaks down the comparisons.

**Table 3:** Pairwise comparisons for classmate knowledgeableness ratings from 1 (Not knowledgeable at all) to 5 (Extremely knowledgeable)

Word	Mean (SD)	Differs from these Words	Does not Differ from these Words
unmodified	3.47 (1.52)	kinda***, kind of*, sorta****, sort of*	absolutely, basically, certainly, clearly, obviously, partially, totally
absolutely	3.52 (1.49)	kinda**, sorta****	unmodified, basically, certainly, clearly, kind of, obviously, partially, sort of, totally
basically	3.48 (1.13)	kinda*, sorta**	unmodified, absolutely, certainly, clearly, kind of, obviously, partially, sort of, totally
certainly	3.65 (1.44)	kinda****, kind of****, sorta****, sort of****	unmodified, absolutely, basically, clearly, obviously, partially, totally
clearly	3.82 (1.06)	kinda****, kind of****, sorta****, sort of****	unmodified, absolutely, basically, certainly, obviously, partially, totally
kinda	3.00 (1.15))	unmodified***, absolutely**, basically*, certainly****, clearly****, obviously****, partially***, totally*,	kind of, sorta, sort of
kind of	3.10 (3)	unmodified*, certainly****, clearly****, obviously**, partially*,	absolutely, basically, kinda, sort of, sorta, totally
obviously	3.69 (1.15)	kinda****, kind of**, sorta****, sort of**	unmodified, absolutely, basically, certainly, clearly, partially, totally
partially	3.60 (1.36)	kinda***, kind of*, sorta****, sort of*	unmodified, obviously, clearly, certainly, basically, totally, absolutely,
sorta	2.81 (1.16)	unmodified****, absolutely****, basically**, certainly****, clearly****, obviously****, partially****, totally****	kinda, kind of, sort of
sort of	3.00 (1.37)	unmodified*, certainly****, clearly****, obviously**, partially*	absolutely, basically, kinda, kind of, sorta, totally
totally	3.43 (1.42)	kinda*, sorta****	unmodified, absolutely, basically, certainly, clearly, kind of, obviously, partially, sort of

Asterisks indicate significance level (* = .05; ** = .01; *** = .001, **** = .0001)

Like in the other two conditions, hedges clustered together and boosters clustered together, with *totally* and *partially* being the odd words out (we note that as with professor and TA knowledgeableness, *basically* clustered with boosters and hedges for classmate knowledgeableness, and differed from only the hedges *kinda* and *sorta*; once again, this result differs from prior studies where *basically* differed from boosters in ratings of negotiation versus telling; see Nguyen & Fox Tree, under review). As with professors, with classmates *totally* did not differ from the unmodified condition and was more knowledgeable than *kinda* and *sorta*. This time *partially* was more knowledgeable than *kinda*, *kind of*, *sorta*, and *sort of*.

#### Summary knowledgeableness

Looking at knowledgeableness ratings across authority levels, most of the low-telling high-negotiation words (hedges) grouped together and most of the high-telling low-negotiation words (boosters) grouped together, with the exceptions of *totally* and *partially*. *Basically* differed from earlier observations where it fell halfway between hedges and boosters on a telling-negotiation scale; across knowledgeabless ratings, *basically* generally clustered with boosters (the exception being that we did not observe differences between *basically* and the hedges *kind of* and *sort of* as used by classmates).

### Friendliness

Across the three authority levels, there was no difference in perceived friendliness in the unmodified condition, *χ*^*2*^(2) = 3.78, *p* = .15. Professors scored an average friendliness score of 3.08 (SD = 1.16), TAs scored an average of 2.80 (SD = 1.04), and classmates scored an average of 2.93 (SD = 1.06).

Looking at the words examined, only three showed significant differences between authority levels. The first was *kind of, χ*^*2*^(2, N = 245) = 7.78, *p* = .02. Post hoc Dunn pairwise comparisons using Bonferroni corrections were carried out. There was no difference between TAs (*m* = 2.98, SD = 1.17) and professors (*m* = 2.93, SD = 1.37) in perceived friendliness when using *kind of*, *p* = 1. There was also no difference between professors and classmates (*m* = 3.40, SD = 1.04) when using *kind of*, *p* = .114. However, classmates who used *kind of* had higher perceived friendliness scores than TAs,* p* = .024. The second word that differed between authority conditions was *totally* (*χ*^*2*^(2, N = 245) = 6.28, *p* = .04), but no post hoc tests were significant. The last word that differed was *clearly* (*χ*^*2*^(2, N = 245) = 6.20, *p* = .045). There were no differences in perceived friendliness between TAs (*m* = 1.55, SD = .899) and professors (*m* = 1.73, SD = 1.10) (*p* > .05) and no differences in perceived friendliness between professors and classmates (*m* = 1.95, SD = 1.09) (*p* > .05). However, classmates were perceived as friendlier than TAs when using *clearly*, *p* = .038. We now examine results within the authority levels.

#### Professor friendliness

Within the student-professor condition (high authority), several words differed from each other, *χ*^*2*^(11) = 246.00, *p* < .001. Pairwise comparisons between groups were carried out. Table 4 breaks down the comparisons.

**Table 4:** Pairwise comparisons for professor friendliness ratings from 1 (Not friendly) to 5 (Extremely friendly)

Word	Mean (SD)	Differs from these Words	Does not Differ from these Words
unmodified	3.08 (1.19)	clearly****, obviously***, totally**	absolutely, basically, certainly, kinda, kind of, partially, sorta, sort of
absolutely	3.23 (1.44)	clearly****, obviously****	unmodified, basically, certainly, kinda, kind of, partially, sorta, sort of, totally
basically	3.24 (1.24)	clearly****, obviously****	unmodified, absolutely, certainly, kinda, kind of, sorta, sort of, partially, totally
certainly	2.99 (1.43)	clearly****, obviously****, totally**	unmodified, absolutely, basically, kinda, kind of, partially, sorta, sort of
clearly	1.73 (1.10)	unmodified****, absolutely****, basically****, certainly****, kinda****, kind of****, partially****, sorta****, sort of****, totally****	obviously
kinda	3.25 (1.16)	clearly****, obviously****	unmodified, absolutely, basically, certainly, kind of, sorta, sort of, partially, totally
kind of	2.93 (1.37)	clearly****, obviously****, totally**	unmodified, absolutely, basically, certainly, kinda, partially, sorta, sort of
obviously	1.67 (1.18)	kind of****, partially****, unmodified****, kinda****, basically****, sort of****, absolutely****, sorta****, totally****, certainly****	clearly
partially	3.12 (.929)	clearly****, obviously****, totally**	unmodified, absolutely, basically, certainly, kinda, kind of, sorta, sort of
sorta	3.31 (1.22)	clearly****, obviously****	unmodified, absolutely, basically, certainly, kinda, kind of, partially, sort of, totally
sort of	3.25 (1.21)	clearly****, obviously****	unmodified, absolutely, basically, certainly, kinda, kind of, partially, sorta, totally
totally	3.77 (1.29)	unmodified**, certainly**, clearly**, kind of**, obviously****, partially**	absolutely, basically, kinda, sorta, sort of

Asterisks indicate significance level (* = .05; ** = .01; *** = .001, **** = .0001)

Two words drove the friendliness ratings: *clearly* and *obviously*. Both lowered the perception of friendliness compared to all other words and the *unmodified* condition. The other boosters (*absolutely* and *certainly*) might have been expected to pattern like *clearly* and *obviously*, but they did not. In fact, *absolutely* and *certainly* did not affect perceived friendliness ratings compared to the *unmodified* condition. *Totally* boosted friendliness ratings compared to *kind of, certainly, clearly, obviously, partially,* and the *unmodified* condition. Unlike with knowledgeableness, however, *partially* did not pattern with *totally*.

#### TA friendliness

Within the student-TA condition (medium authority), several words differed from each other, *χ*^*2*^(11) = 344.1, *p* < .001. Pairwise comparisons between groups were carried out. Table 5 breaks down the comparisons.

**Table 5:** Pairwise comparisons for TA friendliness ratings from 1 (Not friendly) to 5 (Extremely friendly)

Word	Mean (SD)	Differs from these Words	Does not Differ from these Words
unmodified	2.80 (1.04)	clearly****, obviously****, totally**	absolutely, basically, certainly, kinda, kind of, partially, sorta, sort of
absolutely	3.28 (1.02)	clearly****, obviously****	unmodified, basically, certainly, kinda, kind of, sorta, sort of, partially, totally
basically	3.31 (1.02)	clearly****, obviously****	unmodified, absolutely, certainly, kinda, kind of, partially, sorta, sort of, totally
certainly	2.97 (1.11)	clearly****, obviously****, totally**	unmodified, absolutely, basically, kinda, kind of, sorta, sort of, partially
clearly	1.53 (.91)	unmodified****, absolutely****, basically****, certainly****, kinda****, kind of****, partially****, sorta****, sort of****, totally****	obviously
kinda	3.18 (.94)	clearly****, obviously****	unmodified, absolutely, basically, certainly, kind of, partially, sorta, sort of, totally
kind of	2.94 (1.20)	clearly****, obviously****, totally**	unmodified, absolutely, basically, certainly, kinda, partially, sorta, sort of
obviously	1.52 (.94)	unmodified****, absolutely****, basically****, certainly****, kinda****, kind of****, partially****, sorta****, sort of****, totally****	clearly
partially	2.95 (.99)	clearly****, obviously****, totally**	unmodified, absolutely, basically, certainly, kinda, kind of, sorta, sort of
sorta	3.16 (.99)	clearly****, obviously****	unmodified, absolutely, basically, certainly, kinda, kind of, sort of, partially, totally
sort of	3.01 (1.01)	clearly****, obviously****	unmodified, absolutely, basically, certainly, kinda, kind of, partially, sorta, totally
totally	3.45 (1.15)	unmodified**, certainly**, clearly****, kind of**, obviously****, partially**	absolutely, basically, kinda, sorta, sort of

Asterisks indicate significance level (* = .05; ** = .01; *** = .001, **** = .0001)

Like in the professor condition, *clearly* and *obviously* lowered perceived friendliness ratings for TAs compared to using nothing (the *unmodified* condition) or using any of the other words. Additionally, *totally* boosted friendliness ratings compared to using nothing, *kind of, clearly, obviously, partially*, and *certainly*.

#### Classmate friendliness

Within the student-classmate condition (same-level authority), several words differed from each other, *χ*^*2*^(11) = 252.95, *p* < .001. Pairwise comparisons between groups were carried out. Table 6 breaks down the comparisons.

**Table 6:** Pairwise comparisons for classmate friendliness ratings from 1 (Not friendly) to 5 (Extremely friendly)

Word	Mean (SD)	Differs from these Words	Does not Differ from these Words
unmodified	2.93 (1.36)	clearly****,obviously****	absolutely, basically, certainly, kinda, kind of, partially, sorta, sort of, totally
absolutely	3.01 (1.35)	clearly****, obviously****	unmodified, basically, certainly, kinda, kind of, partially, sorta, sort of, totally
basically	3.46 (1.06)	clearly****, obviously****, partially*	unmodified, absolutely, certainly, kinda, kind of, sorta, sort of, totally
certainly	2.93 (1.33)	clearly****, obviously****	unmodified, absolutely, basically, kinda, kind of, partially, sort of, sorta, totally
clearly	1.95 (1.09)	unmodified****, absolutely****, kinda****, sorta****, kind of****, totally****, basically****, partially****, certainly****, sort of****	obviously
kinda	3.34 (1.04)	clearly****, obviously****	unmodified, absolutely, basically, certainly, kind of, sorta, sort of, partially, totally
kind of	3.40 (1.06)	clearly****, obviously****	unmodified, absolutely, basically, certainly, kinda, sorta, sort of, partially, totally
obviously	1.48 (.955)	unmodified****, absolutely****, basically****, certainly****, kinda****, kind of****, partially****, sorta****, sort of****, totally****	clearly
partially	2.92 (1.23)	basically*, clearly****, obviously****	unmodified, absolutely, certainly, kinda, kind of, sorta, sort of, totally
sorta	3.30 (1.26)	clearly****, obviously****	unmodified, absolutely, basically, certainly, kinda, kind of, partially, sort of, totally
sort of	3.07 (1.25)	clearly****, obviously****	unmodified, absolutely, basically, certainly, kinda, kind of, partially, sorta, totally
totally	3.36 (1.42)	clearly****, obviously****	unmodified, absolutely, basically, certainly, kinda, kind of, partially, sorta, sort of

Asterisks indicate significance level (* = .05; ** = .01; *** = .001; **** = .0001)

Like in the high-authority and medium-authority conditions, *clearly* and *obviously* had the greatest negative impact on perceived friendliness. No other words were significantly different from the *unmodified* condition.

#### Summary friendliness

Across authority conditions, *clearly* and *obviously* lowered friendliness ratings. Two other boosters, *absolutely* and *certainly*, did not affect ratings. In the high- and medium-authority conditions (professor and TA), *totally* was friendlier than the *unmodified* condition. However, in the classmate group, only *clearly* and *obviously* (but not *totally*) differed from the unmodified condition. So, *totally* can buy friendliness, but only from communicators with greater authority.

### Politeness

In the unmodified condition, across the three levels of authority there were no differences in perceived politeness, *χ*^*2*^(2) = 2.30, *p* = .31.

Only one word differed in perceived politeness between the three conditions, *clearly*, *χ*^*2*^(2) = 8.45, *p* = .015. Professors who used *clearly* (*m* = 2.03, SD = 1.2) had no difference in politeness compared to classmates (m = 2.12, SD = 1.10), *p* = .109, or TAs (*p* = 1). However, TAs who used *clearly* (m = 1.68, SD = 1.00) were seen as less polite than classmates (*p* = .017).

#### Professor politeness

A nonparametric Friedman’s test was carried out. Within the student-professor condition (high authority), there was a significant difference in the underlying distributions of each word, *χ*^*2*^(11) = 222.17, *p* < .001. Pairwise Dunn–Bonferroni comparisons between words were carried out, with Bonferroni corrections applied. Table 7 breaks down the comparisons and shows which words differ from one another.

**Table 7:** Pairwise comparisons for professor politeness ratings from 1 (Not polite) to 5 (Extremely polite)

Word	Mean (SD)	Differs from these Words	Does not Differ from these Words
unmodified	3.43 (1.13)	clearly****, obviously*****	absolutely, basically, certainly, kinda, kind of, partially, sorta, sort of, totally
absolutely	3.23 (1.37)	clearly****, obviously****	unmodified, basically, certainly, kinda, kind of, sorta, sort of, partially, totally
basically	3.19 (1.26)	clearly****, obviously****	unmodified, absolutely, certainly, kinda, kind of, partially, sorta, sort of, totally
certainly	3.21 (1.45)	clearly****, obviously****	unmodified, absolutely, basically, kinda, kind of, partially, sorta, sort of, totally
clearly	2.03 (1.23)	unmodified****, absolutely****, basically****, certainly****, kinda****, kind of****, partially****, sorta****, sort of****, totally****	obviously
kinda	3.07 (1.23)	clearly****, obviously****	unmodified, absolutely, basically, certainly, kind of, partially, sorta, sort of, totally
kind of	2.95 (1.39)	clearly****, obviously****	unmodified, absolutely, basically, certainly, kinda, partially, sorta, sort of, totally
obviously	1.72 (1.27)	unmodified****, absolutely****, basically****, certainly****, kinda****, kind of****, sorta****, sort of****, partially****, totally****	clearly
partially	3.23 (1.02)	clearly****, obviously****	unmodified, absolutely, basically, certainly, kinda, kind of, sorta, sort of, totally
sorta	3.08 (1.22)	clearly****, obviously****	unmodified, absolutely, basically, certainly, kinda, kind of, partially, sort of, totally
sort of	3.20 (1.11)	clearly****, obviously****	unmodified, absolutely, basically, certainly, kinda, kind of, partially, sorta, totally
totally	3.31 (1.22)	clearly****, obviously****	unmodified, absolutely, basically, certainly, kinda, kind of, partially, sorta, sort of

Asterisks indicate significance level (* = .05; ** = .01; *** = .001, **** = .0001)

*Clearly* and *obviously* lowered perceived politeness compared to using nothing (the *unmodified* condition) and the rest of the words tested. No other words had an effect compared to the *unmodified* condition or compared to each other.

#### TA politeness

A nonparametric Friedman’s test was carried out. Within the student-TA condition (medium authority), there was a significant difference in the underlying distributions of each word, *χ*^*2*^(11) = 347.29, *p* < .001. Pairwise Dunn–Bonferroni comparisons between words were carried out, with Bonferroni corrections applied. Table 8 breaks down the comparisons and shows which words differ from one another.

**Table 8:** Pairwise comparisons for TA politeness ratings from 1 (Not polite) to 5 (Extremely polite)

Word	Mean (SD)	Differs from these Words	Does not Differ from these Words
unmodified	3.31 (.93)	clearly****, obviously****	absolutely, basically, certainly, kinda, kind of, partially, sorta, sort of, totally
absolutely	3.23 (.92)	clearly****, obviously****	unmodified, basically, certainly, kinda, kind of, partially, sorta, sort of, totally
basically	3.29 (.94)	clearly****, obviously****	unmodified, absolutely, certainly, kinda, kind of, partially, sorta, sort of, totally
certainly	3.14 (1.03)	clearly****, obviously****	unmodified, absolutely, basically, kinda, kind of, partially, sort of, sorta, totally
clearly	1.68 (1.00)	unmodified****, absolutely****, basically****, certainly****, kinda****, kind of****, sorta****, sort of****, partially****, totally****	obviously
kinda	3.07 (.94)	obviously****, clearly****	unmodified, absolutely, basically, certainly, kind of, partially, sorta, sort of, totally
kind of	3.11 (1.20)	obviously****, clearly****	unmodified, absolutely, basically, certainly, kinda, partially, sorta, sort of, totally
obviously	1.36 (.84)	sort of****, sorta****, kinda****, kind of****, totally****, absolutely****, basically****, partially****, unmodified****, certainly****	clearly
partially	3.29 (.93)	clearly****, obviously****	unmodified, absolutely, basically, certainly, kinda, kind of, sort of, sorta, totally
sorta	3.09 (.91)	clearly****, obviously****	unmodified, absolutely, basically, certainly, kinda, kind of, partially, sort of, totally
sort of	3.00 (1.00)	clearly****, obviously****	unmodified, absolutely, basically, certainly, kinda, kind of, sorta, partially, totally
totally	3.16 (1.03)	clearly****, obviously****	unmodified, absolutely, basically, certainly, kinda, kind of, partially, sorta, sort of

Asterisks indicate significance level (* = .05; ** = .01; *** = .001, **** = .0001)

*Clearly* and *obviously* again lowered politeness ratings compared to the other words as well as compared to the *unmodified* condition.

#### Classmate politeness

A nonparametric Friedman’s test was carried out. Within the student-professor condition (same-level authority), there was a significant difference in the underlying distributions of each word, *χ*^*2*^(11) = 314.36, *p* <.001. Pairwise Dunn–Bonferroni comparisons between words were carried out, with Bonferroni corrections applied. Table 9 breaks down the comparisons and shows which words differ from one another.

**Table 9:** Pairwise comparisons for classmate politeness ratings from 1 (Not polite) to 5 (Extremely polite)

Word	Mean (SD)	Differs from these Words	Does not Differ from these Words
unmodified	3.13 (1.38)	clearly****, obviously****	absolutely, basically, certainly, kinda, kind of, partially, sorta, sort of, totally
absolutely	2.92 (1.38)	clearly****, obviously****	unmodified, basically, certainly, kinda, kind of, partially, sorta, sort of, totally
basically	3.22 (1.01)	clearly****, obviously****	unmodified, absolutely, certainly, kinda, kind of, sorta, sort of, partially, totally
certainly	3.00 (1.38)	clearly****, obviously****	unmodified, absolutely, basically, kinda, kind of, partially, sorta, sort of, totally
clearly	2.12 (1.11)	unmodified****, absolutely****, basically****, certainly****, kinda****, kind of****, partially****, sorta****, sort of****, totally****	obviously
kinda	3.18 (1.06)	clearly****, obviously****	unmodified, absolutely, basically, certainly, kind of, partially, sorta, sort of, totally
kind of	3.34 (1.00)	clearly****, obviously****	unmodified, absolutely, basically, certainly, kinda, partially, sorta, sort of, totally
obviously	1.60 (1.02)	unmodified****, absolutely****, basically****, certainly****, kinda****, kind of****, partially****, sorta****, sort of****, totally****,	clearly
partially	3.11 (1.22)	clearly****, obviously****	unmodified, absolutely, basically, certainly, kinda, kind of, sorta, sort of, totally
sorta	3.20 (1.11)	clearly****, obviously****	unmodified, absolutely, basically, certainly, kinda, kind of, partially, sort of, totally
sort of	3.02 (1.16)	clearly****, obviously****	unmodified, absolutely, basically, certainly, kinda, kind of, partially, sorta, totally
totally	3.04 (1.34)	clearly****, obviously****	unmodified, absolutely, basically, certainly, kinda, kind of, partially, sorta, sort of

Asterisks indicate significance level (* = .05; ** = .01; *** = .001, **** = .0001)

As in the other conditions, *clearly* and *obviously* lowered politeness ratings compared to the other words as well as compared to the *unmodified* condition.

#### Summary politeness

Across authority conditions, *clearly* and *obviously* lowered politeness ratings.

### Professionalism

There were significant differences between professionalism ratings and authority conditions. Starting with the unmodified condition, there was a significant difference in how professional professors, TAs, and classmates were perceived, *χ*^*2*^(2) = 17.89, *p* < .001. Following this with pairwise Dunn tests with Bonferroni corrections, when answering the question in the *unmodified* condition, classmates (*m* = 3.55, SD = 1.54) were perceived as less professional than TAs (*m* = 4.29, SD = 1.03), *p* < .001, and less professional than professors (*m* = 4.25, SD = 1.19), *p* < .001. There was no difference between how TAs and professors were perceived professionally (*p* = 1) in the *unmodified* condition.

There was also a significant difference in how *basically* affected professionalism ratings across conditions, *χ*^*2*^(2) = 14.19, *p* < .001. Pairwise comparisons show that there was no difference between TAs (*m* = 3.67, SD = .97) and professors (*m* = 3.48, SD = 1.12) in ratings (*p* = 1). However, when using *basically* classmates (*m* = 3.17, SD = 1.01) were perceived as less professional than professors (*p* = .003) and less professional than TAs (*p* = .004).

*Absolutely* also differed between conditions, *χ*^*2*^(2) = 6.81, *p* < .05. Pairwise comparisons show that there was no significant difference between how professors (*m* = 3.55, SD = 1.54) and TAs (*m* = 3.82, SD = .95) were rated (*p* = 1). There was also no significant difference between how classmates (*m* = 3.28, SD = 1.40) and professors were rated (*p* = .127). However, there was a significant difference between how TAs and classmates were perceived, with classmates rated as less professional than TAs when using *absolutely* (*p* = *.04*).

*Clearly* also differed between conditions, *χ*^*2*^(2) = 15.29, *p* < .001. Professors (*m* = 2.56, SD = 1.47) and TAs (*m* = 2.22, SD = 1.19) did not differ on ratings of perceived professionalism when using *clearly*, (*p* = .258). Neither did professors or classmates (*m* = 3.04, SD = 1.41) when using *clearly* (*p* = .117). However, TAs were scored lower on professionalism when using *clearly* in the response, compared to classmates (*p* < .001).

#### Professor professionalism

A nonparametric Friedman’s test was carried out. Within the student-professor condition (high authority), there was a significant difference between words, *χ*^*2*^(11) = 212.44, *p* < .001. Pairwise Dunn–Bonferroni comparisons between words were carried out, with Bonferroni corrections applied. Table 10 breaks down the comparisons and shows which words differ from one another.

**Table 10:** Pairwise comparisons for professor politeness ratings from 1 (Extremely unprofessional) to 5 (Extremely professional)

Word	Mean (SD)	Differs from these Words	Does not Differ from these Words
unmodified	4.24 (1.19)	certainly****, clearly****, kinda****, kind of****,obviously****, sorta****, sort of****, totally****	absolutely, basically, partially
absolutely	3.55 (1.54)	clearly****, kinda***, obviously****, sorta***	unmodified, basically, certainly, kind of, partially, sort of, totally
basically	3.60 (1.40)	clearly****, kinda***, obviously****, sorta***	unmodified, absolutely, certainly, kind of, partially, sort of, totally
certainly	3.31 (1.46)	unmodified****, clearly**, obviously****	absolutely, basically, kinda, kind of, partially, sorta, sort of, totally
clearly	2.56 (1.47)	unmodified****, absolutely****, basically****, certainly**, partially****	kinda, kind of, obviously, sorta, sort of, totally
kinda	2.81 (1.322)	unmodified****, absolutely***, basically***, partially****	certainly, clearly, kind of, obviously, sorta, sort of, totally
kind of	2.95 (1.46)	unmodified****, obviously*, partially*	absolutely, basically, certainly, clearly, kinda, sorta, sort of, totally
obviously	2.25 (1.46)	unmodified****, absolutely****, basically****, certainly****, kind of*, partially****, sort of***, totally*	clearly, kinda, sorta
partially	3.83 (1.21)	clearly****, kinda****, kind of*, obviously****, sorta****, totally*,	unmodified, absolutely, basically, certainly, sort of
sorta	2.79 (1.29)	unmodified****, absolutely***, basically***, partially****	certainly, clearly, kinda, kind of, obviously, sort of, totally
sort of	3.17 (1.31)	unmodified****, obviously***	absolutely, basically, certainly, clearly, kinda, kind of, partially, sorta, totally
totally	3.08 (1.31)	unmodified****, obviously*, partially*	absolutely, basically, certainly, clearly, kinda, kind of, sorta, sort of

Asterisks indicate significance level (* = .05; ** = .01; *** = .001, **** = .0001)

Most words lowered perceived professionalism. *Obviously* lowered perceived professionalism more than seven words and the unmodified condition. The professionalism of *obviously* was similar to the professionalism of *clearly*, *kinda*, and *sorta*.

Only three words did not lower the perceived professionalism compared to the *unmodified* condition: *absolutely, basically,* and *partially*. The measures of central tendency for the *unmodified* condition were close to the top of the rating scale (median = 4, mean = 4.25, mode = 5). This suggests that the participants had a default assumption that a professor will be professional.

#### TA professionalism

A nonparametric Friedman’s test was carried out. Within the student-TA condition (medium authority), there was a significant difference between words, *χ*^*2*^(11) = 331.52, *p* < 001. Pairwise Dunn–Bonferroni comparisons between words were carried out, with Bonferroni corrections applied. Table 11 breaks down the comparisons and shows which words differ from one another.

**Table 11:** Pairwise comparisons for TA politeness ratings from 1 (Extremely unprofessional) to 5 (Extremely professional)

Word	Mean (SD)	Differs from these Words	Does not Differ from these Words
unmodified	4.29 (1.04)	clearly****,kinda****, kind of****, obviously****, sorta****, sort of****, totally****	absolutely, basically, certainly, partially
absolutely	3.82 (.95)	clearly****, kinda****, kind of*, obviously****, sorta****, sort of***, totally****	unmodified, basically, certainly, partially
basically	3.67 (.97)	clearly****, kinda**, obviously****, sorta****, sort of*, totally**	unmodified, absolutely, certainly, kind of, partially
certainly	3.80 (1.14)	clearly****, kinda****, kind of*, obviously****, sorta****, sort of****, totally****	unmodified, absolutely, basically, partially
clearly	2.22 (1.20)	unmodified****, absolutely****, basically****, certainly****, kind of**, partially****	kinda, obviously, sorta, sort of, totally
kinda	2.98 (1.09)	unmodified****, absolutely****, basically**, certainly****, obviously****, partially**	clearly, kind of, sorta, sort of, totally
kind of	3.13 (1.33)	unmodified****, absolutely*, certainly*, clearly**, obviously****	basically, kinda, partially, sorta, sort of, totally
obviously	1.91 (1.05)	unmodified****, absolutely****, basically****, certainly****, kinda****, kind of****, partially****, sorta**, sort of****, totally****	clearly
partially	3.61 (1.25)	clearly****, kinda**, obviously****, sorta****, sort of*, totally**	unmodified, absolutely, basically, certainly, kind of
sorta	2.77 (1.13)	unmodified****, absolutely****, basically****, certainly****, obviously**, partially****	clearly, kinda, kind of, sort of, totally
sort of	3.02 (1.16)	unmodified****, absolutely***, basically*, certainly****, obviously****, partially*	clearly, kinda, kind of, sorta, totally
totally	2.98 (1.18)	unmodified****, absolutely****, basically**, certainly****, obviously****, partially**	clearly, kinda, kind of, sorta, sort of

Asterisks indicate significance level (* = .05; ** = .01; *** = .001, **** = .0001)

Once again, most words lowered perceived professionalism. *Obviously* lowered perceived professionalism more than nine words and the unmodified condition. The professionalism of *obviously* was similar to the professionalism of *clearly*.

Only four words did not lower the perceived professionalism compared to the *unmodified* condition: *absolutely, basically, certainly*, and *partially*. The measures of central tendency for the *unmodified* condition were close to the top of the rating scale (median = 5.00, mean = 4.29, mode = 5). This suggests that the participants had a default assumption that a TA will be professional.

#### Classmate professionalism

A nonparametric Friedman’s test was carried out. Within the student-classmate condition (same-level authority), there was a significant difference between words, *χ*^*2*^(11) = 145.71, *p* < 001. Pairwise Dunn–Bonferroni comparisons between words were carried out, with Bonferroni corrections applied. Table 12 breaks down the comparisons and shows which words differ from one another.

**Table 12:** Pairwise comparisons for classmate politeness ratings from 1 (Extremely unprofessional) to 5 (Extremely professional)

Word	Mean (SD)	Differs from these Words	Does not Differ from these Words
unmodified	3.59 (1.59)	kinda*, obviously****, sort of****, totally***	absolutely, basically, certainly, clearly, kind of, partially, sorta
absolutely	3.28 (1.40)	obviously****, sorta*	unmodified, basically, certainly, clearly, kinda, kind of, partially, sort of, totally
basically	3.17 (1.08)	*-*	unmodified, absolutely, certainly, clearly, kinda, kind of, obviously, partially, sorta, sort of, totally
certainly	3.51 (1.53)	clearly*, kinda**, obviously****, sorta****, sort of*, totally****	unmodified, absolutely, basically, kind of, partially
clearly	3.04 (1.41)	certainly*, obviously**, partially**	unmodified, absolutely, basically, kinda, kind of, sorta, sort of, totally
kinda	2.95 (1.20)	unmodified*, certainly**, obviously*, partially***	absolutely, basically, clearly, kind of, sorta, sort of, totally
kind of	3.31 (1.40)	obviously****	unmodified, absolutely, basically, certainly, clearly, kinda, partially, sorta, sort of, totally
obviously	2.19 (1.27)	unmodified****, basically****, absolutely****, certainly****, clearly**, kinda*, kind of****, partially****, sort of**	sorta, totally
partially	3.60 (1.45)	clearly**, kinda***, obviously****, sorta****, sort of**, totally****	unmodified, absolutely, basically, certainly, kind of
sorta	2.92 (1.38)	certainly*, obviously**, partially**	unmodified, absolutely, basically, clearly, kinda, kind of, sorta, totally
sort of	2.71 (1.21)	unmodified****, absolutely*, certainly****, partially****	basically, clearly, kinda, kind of, obviously, sort of, totally
totally	2.70 (1.09)	unmodified***, certainly****, partially****	absolutely, basically, clearly, kinda, kind of, obviously, sorta, sort of

Asterisks indicate significance level (* = .05; ** = .01; *** = .001, **** = .0001)

Once again, most words lowered perceived professionalism. *Obviously* lowered perceived professionalism more than eight words and the unmodified condition. The professionalism of *obviously* was similar to the professionalism of *sorta* and *totally*.

Unlike with higher authority professors and TAs, most words did not lower perceived professionalism of classmates compared to the *unmodified* condition. The only words that lowered professionalism were *kinda, obviously*, *sort of* and *totally*.

Like the other levels of authority, the measures of central tendency for the *unmodified* condition were close to the top of the rating scale (median = 4.00, mean = 3.55, mode = 5). This suggests that the participants had a default assumption that classmates will be professional.

#### Summary professionalism

For professors and TAs, most words lowered perceived professionalism. For classmates, most words did not lower perceived professionalism. For the professors and TAs, similar words did not lower professionalism: *absolutely, basically, certainly* (TAs only), and *partially*. For the classmates, the set of words that lowered professionalism included hedges and boosters: *kinda*, *obviously*, *sort of*, and *totally*. There was a default assumption of professionalism across all levels of authority.

## Discussion

Across assessments of knowledgeableness, friendliness, politeness, and professionalism, the greatest distinction between authority levels (professor, TA, classmate) was on professionalism. For professors and TAs, most negotiation words lowered professionalism. In contrast, for classmates, most negotiation words did not lower professionalism. Ratings of professionalism were fairly high across all authority levels, however. The greatest similarity in assessments was for friendliness and politeness. Across all authority conditions, *clearly* and *obviously* made people appear less friendly and less polite. The other negotiation words were similar to each other. The result that was the most similar to prior findings (Nguyen & Fox Tree, under review) was those related to knowledgeableness. While these ratings did not differ by authority level, snippets with hedges were rated as less knowledgeable than the unmodified condition. At the same time, snippets with boosters were not more rated as more knowledgeable than unmodified snippets. A surprising finding was that *totally* was rated as similar to the hedges *kind of* and *sort of* (but differed from *kinda* and *sorta*). Totally is typically treated as a booster and should have, technically, differed from all hedges. An additional surprising finding about *totally* was that it helped higher authority speakers (professors and TAs but not classmates) appear more friendly compared to no negotiation word.

There are many phrases that can act in both literal and pragmatic ways, such as *I don’t know*, which can convey not having the information as well as the social meaning of being uncertain about the information (Nguyen & Fox Tree, 2025). Like *I don’t know*, *totally* carries many pragmatic interpretations, some of which may have very strong effects on speaker perception, such as age. As noted in Beltrama (2018), *totally* has a literal use conveying completeness as well as a pragmatic use managing common ground—speakers can use *totally* to mark information that should be added to shared mutual understanding. We now turn to discussion of how negotiation words affect social evaluation of knowledgeableness, friendliness, politeness, and professionalism.

### Knowledgeableness

We predicted that hedges would decrease perceived ratings of knowledgeableness, and this is what we found. For evaluating the perceived knowledge of a speaker, hedges really do act as hedges. Regardless of authority level, hedges lowered the rating of perceived knowledge compared to the unmodified conditions, suggesting that when people are attempting to determine how much to trust the speaker, they do take hedges into account as markers of a lack of knowledge. Conversely, and contrary to our predictions, words that are thought of as boosters did *not* boost perceived knowledge of a speaker compared to unmodified speech. While they did cluster together in how they related to other words, they did not make a speaker appear more knowledgeable. Said another way, we hypothesized that these words would influence social perceptions of knowledgeableness, but we did not find that to be the case. This suggests that when it comes to judging how informed a speaker is, addressees use hedges and boosters differently as sources of evidence. While hedges can be taken as evidence that the speaker is uninformed, boosters are not taken as evidence that the speaker is well-informed. It is likely that in these cases, while both are metalinguistic commentaries, the words categorized as boosters indicate something else about the speaker (perhaps assertiveness) rather than how knowledgeable they are.

### Friendliness, politeness, and professionalism

With respect to friendliness and politeness, two words stood out. *Clearly* and *obviously* lowered both friendliness and politeness ratings. Because neither word had an effect on perceived knowledge ratings, but did affect perceived friendliness and politeness ratings, we conclude that *clearly* and *obviously* act as metalinguistic commentary on the relationship between the speaker and the addressee (friendliness and politeness are expressed across conversational participants) rather than as commentary on the speaker themselves (such as the speaker’s knowledge, which is a manifestation of an individual participant’s characteristics). Additional evidence is provided by looking across authority levels. In our study, *clearly* was most rude when coming from a TA, providing further evidence that the relationship between communicators is important to understanding what some negotiation words imply.

For perceived professionalism, classmates were given the lowest ratings of perceived professionalism when the answer was unmodified. Classmates were also the least likely to be penalized for using words that might be deemed less professional if they were coming from a professor or a TA. This suggests that people might be more generous to their peers in evaluations of professionalism compared to people who should hold authority over them.

Returning to our predictions about friendliness, politeness, and professionalism, we predicted that boosters would increase ratings of professionalism and that hedges would increase ratings of friendliness and politeness. However, this is not what we found. Most negotiation words did not affect ratings of friendliness, politeness, and professionalism. Only two boosters, *clearly* and *obviously*, affected ratings with both lowering friendliness and politeness. For professionalism, authority played a role in how people were perceived. For those in the high- and mid-level-authority conditions, almost all words lowered professionalism.

### Formality

The inherent formality level of a word was less important than whether the word was a booster or hedge. For example, with respect to the two hedges *kinda* and *partially* in Table 2 on TA knowledgeableness, *kinda* (informal) did not differ from *partially* (formal). And with respect to the two boosters *totally* and *absolutely* in Table 2, *totally* (informal) did not differ from *absolutely* (formal). Studies of the use of these words in other contexts may find differences in formality however. Indeed, we found some differences ourselves; in Table 11 on TA professionalism, *kinda* differed from *partially* and *totally* differed from *absolutely* (although Table 10 on professor professionalism and Table 12 on classmate professionalism do not show the *totally*/*absolutely* distinction). We did not treat formality of words as a predictor variable in the current study because the frame of the study was an informal context (mock Discord). The tables we provide may help other researchers develop hypotheses about word formality however, including hypotheses about how formality affects social variables across formal and informal contexts.

## Conclusion

In the current report, we focused on how negotiation words interacted with sociolinguistic features to support the grounding process. While it is well understood that people use multiple cues beyond the words themselves to reach mutual understanding, little work has been done on how explicit cues to negotiation interact with features of the people using the cues in dyadic conversations. Previous researchers have also observed a relationship between negotiation words and expressions of attitudes, in experiments looking at non-dialogue stimuli (such as *totally* as a speaker-oriented modifier; see Beltrama, 2018). Expanding that research to look at dialogues is important for building better models of human communication.

We found that negotiation words did affect impressions of authority. When reading negotiation words, people must decide what the speaker intended; for example, were speakers trying to convey authority, or were they trying to do something else? People may wish to appear lower or higher in authority for many reasons; for example, appearing lower in authority may put addressees at ease, while appearing higher in authority might increase speakers’ credibility. It is the job of the listener to integrate the words used with their understanding of the social context and what the baseline rate of authority for the speaker should be. Thus, it is important to understand how negotiation words can convey authority—or a lack of authority.

One arena where this is highly relevant is in education. Instructors’ informal language has been linked to learning improvements (Moreno & Mayer, 2000, 2004; Schneider, 2015). There may be benefits to instructors for using the right negotiation words as well. With the right negotiation words, instructors may appear more casual and approachable, which can create more equitable classroom settings. Positive interactions with professors, teaching assistants, and classmates can support learning and make students feel comfortable asking for and receiving help, both in-person (Holley & Steiner, 2005; Ortiz, 1988) and online (Kim et al., 2023; see Frisby et al., 2014, and Micari & Calkins, 2021, for discussion of how instructors can foster a positive space for help-seeking behavior). Understanding how words build community in online spaces is vital as we move into a digital learning world. Classes with an online component took off after the pandemic and are likely to remain a large part of education; it is critical to understand how people interact in these spaces.

Another arena where informal written communication is highly relevant is online discussion groups. People share information online in a variety of ways, from posts on community forums or social media sites to comments sections of newspapers. Understanding how negotiation words intersect with authority impressions is especially important when it comes to understanding how political viewpoints are shaped and how authoritarian figures rise and gain power in online spaces. People may adopt personas that suggest authority they do not have, simply by choosing words that imply certainty and knowledge, or they may adopt personas that convey friendliness and politeness as a way to gain trust with their audience.

Future researchers can expand on this work by testing a wider variety of participants in a wider range of settings. Almost all of the participants in this experiment were young adults raised in California who spoke California English as a first language. Norms for what is considered proper speech to use by this group, and in the academic setting we tested, may not be the same for other groups or other settings. For example, we know that norms are shaped by culture (both at the individual level and at the societal level) and that norms differ across speakers of different varieties of English (Schneider & Placencia, 2017). As people increasingly communicate in textual formats for a wide variety of professional and personal reasons, it is increasingly important to take a close look at the negotiation words people use.

## Data Availability

Release of participant data was not obtained at the time data was collected; therefore, data are not available publicly.
